# Whole-Genome Sequencing and Annotation of Fibrobacter succinogenes HC4, Isolated from the Horse Cecum

**DOI:** 10.1128/mra.00440-22

**Published:** 2022-10-13

**Authors:** Alicia Froidurot, Emmanuel Jacotot, Véronique Julliand

**Affiliations:** a Université Bourgogne-Franche-Comté, L’Institut Agro Dijon, PAM UMR A 02.102, Dijon, France; University of Maryland School of Medicine

## Abstract

Fibrobacter succinogenes is a major cellulolytic bacterial species living in the large intestines of herbivores. This study reports the genome sequencing, assembly, and annotation of F. succinogenes HC4 (DSM 33656), a strain isolated from horse cecal contents. The genome comprised a total of 3.74 Mbp, with a G+C content of 48.96%.

## ANNOUNCEMENT

Fibrobacter succinogenes plays a key role in host nutrition and health by degrading cellulose into short-chain fatty acids (SCFAs) (1–5) in the digestive ecosystem. To date, cultured representatives of F. succinogenes living in the large intestines of mammals are lacking.

To isolate cellulolytic strains of F. succinogenes, samples were collected from one cecum-cannulated horse (6). Contents were stored at 38°C in CO_2_-saturated containers. Decimal dilutions were inoculated in a specific medium (7, 8). After 7 days of incubation at 38°C under CO_2_, the roll tube method (9) and the enrichment method (10) were used alternately to isolate strain HC4. The 16S rRNA gene was amplified by PCR using the universal primers 27f and 1492r. DNA amplification was achieved using the following program: denaturation for 4 min at 95°C; 35 cycles consisting of 1 min at 94°C, 1 min at 55°C, and 2 min at 72°C; and then 15 min at 72°C and 3 min at 30°C (11). PCR products were sequenced by Genewiz using the Sanger method. The 16S rRNA gene of strain HC4 (accession number OP018198) exhibited 99% sequence identity to those of F. succinogenes group V equine strains (10). For genome sequencing, DNA was extracted from a 48-h culture of F. succinogenes HC4 at 38°C under CO_2_ in Lowe medium (12) containing cellulose filter paper (13). The culture sample was concentrated and subjected to physical lysis using a bead beater (3 min at 25 Hz) and chemical lysis using a lysis buffer. After centrifugation for 5 min at 16,000 × *g* and treatment with 10 M ammonium acetate and 70% (v/v) ethanol, DNA was purified using the QIAamp stool minikit from Qiagen (catalog number 51504). The F. succinogenes genome was sequenced, assembled, and annotated by Genewiz. Libraries were constructed using the NEBNext Ultra II DNA library preparation kit for Illumina and sequenced using an Illumina NovaSeq sequencing platform (2 × 150 bp). A total of 13,163,761 raw reads were obtained and filtered with bcl2fastq v2.20.0 (14). Sequences of <30 bp were discarded. *De novo* assembly, with 50 scaffolds with an *N*_50_ value of 149,151 bp, was carried out using SPAdes v3.10.0 (k-mer values of 21, 33, 55, and 77) (15). The *de novo* assembled genome was created with a minimum contig length of 1,000 bp and analyzed statistically using QUAST v4.6 (16). Gene prediction and annotation were performed using Prokka v1.12 (17) with ISfinder, the NCBI Bacterial Antimicrobial Resistance Reference Gene Database, and the UniProtKB database. Default parameters were used for all software unless otherwise specified. The F. succinogenes HC4 genome comprised a total of 3.74 Mbp, with 3,143 coding sequences. The G+C content of the genome was 48.96%.

The HC4 strain of F. succinogenes will allow better characterization of the physiology and ecology of cellulolytic bacteria inhabiting the large intestines of mammals.

### Data availability.

This whole-genome shotgun project has been deposited in DDBJ/ENA/GenBank under the accession number JALCZV000000000 (BioProject accession number PRJNA812370), and the raw reads have been deposited in the NCBI Sequence Read Archive (SRA) under the SRA accession number SRR18517561. The version described in this paper is version JALCZV010000000. The sequence of the 16S rRNA gene of strain HC4 has been deposited under accession number OP018198.
